# Converging pathways involving microRNA-206 and the RNA-binding protein KSRP control post-transcriptionally utrophin A expression in skeletal muscle

**DOI:** 10.1093/nar/gkt1350

**Published:** 2013-12-26

**Authors:** Adel Amirouche, Helina Tadesse, Pedro Miura, Guy Bélanger, John A. Lunde, Jocelyn Côté, Bernard J. Jasmin

**Affiliations:** ^1^Department of Cellular and Molecular Medicine, University of Ottawa, Ottawa, Ontario K1H 8M5, Canada and ^2^Centre for Neuromuscular Disease, University of Ottawa, Ottawa, Ontario K1H 8M5, Canada

## Abstract

Several reports have previously highlighted the potential role of miR-206 in the post-transcriptional downregulation of utrophin A in cultured cells. Along those lines, we recently identified K-homology splicing regulator protein (KSRP) as an important negative regulator in the post-transcriptional control of utrophin A in skeletal muscle. We sought to determine whether these two pathways act together to downregulate utrophin A expression in skeletal muscle. Surprisingly, we discovered that miR-206 overexpression in cultured cells and dystrophic muscle fibers causes upregulation of endogenous utrophin A levels. We further show that this upregulation of utrophin A results from the binding of miR-206 to conserved sites located in the 3′-UTR (untranslated region) of KSRP, thus causing the subsequent inhibition of KSRP expression. This miR-206-mediated decrease in KSRP levels leads, in turn, to an increase in the expression of utrophin A due to a reduction in the activity of this destabilizing RNA-binding protein. Our work shows that miR-206 can oscillate between direct repression of utrophin A expression via its 3′-UTR and activation of its expression through decreased availability of KSRP and interactions with AU-rich elements located within the 3′-UTR of utrophin A. Our study thus reveals that two apparent negative post-transcriptional pathways can act distinctively as molecular switches causing repression or activation of utrophin A expression.

## INTRODUCTION

MicroRNAs (miRNAs) constitute a class of small non-coding RNAs that are 20–23 nt in length and are evolutionarily conserved (1). Binding of a miRNA to its target can occur by perfect or imperfect base pairing at the seed sequence, resulting in translational inhibition or messenger RNA (mRNA) degradation (2,3). MiRNAs have been implicated in the regulation of the skeletal muscle phenotype, through the modulation of transcription factors and other signaling molecules involved in skeletal muscle cell proliferation and differentiation as well as muscle regeneration (4–6). Several chronic disorders including neuromuscular diseases such as Duchenne muscular dystrophy (DMD), facioscapulohumeral muscular dystrophy, myotonic dystrophy type 1, limb-girdle muscular dystrophies types 2A and 2B, Miyoshi myopathy and inclusion body myositis, all have in common disruption in the pattern of expression of miRNAs (7–10).

MiR-206, also referred to as myomiR-206, is unique in its skeletal muscle-specific expression pattern (11,12). The miR-206 gene is located between the polycystic kidney and hepatic disease 1 gene and the interleukin-17 gene in mouse (chr 1), rat (chr 9) and human (chr 6) (5,11,13). The expression of miR-206 can be detected during mouse embryonic development at a low level and as early as 9.5 days post-coitum (dpc). It then significantly increases thereafter and is thought to be critical for proper myogenic differentiation (14). MicroRNA-206 contains two promoters, the proximal promoter responsible for the transcription of miR-206, and the distal one driving expression of the whole transcript containing miR-133 b, miR-206 and a long non-coding RNA (15). The transcription factors MyoD and myogenin bind to the upstream regions of miR-206, and are thus likely to regulate its expression (16,17). During myogenic differentiation, miR-206 is believed to control the balance between differentiation and proliferation of skeletal muscle cells (5,11). *In vivo*, miR-206 has also been shown to regulate skeletal muscle regeneration (18–20). In this context, miR-206 is capable of regulating Pax3 (21), Pax7 (22), HDAC4 (23) Notch3 (24) and BDNF (25) expression, all essential components involved in muscle development. Finally, several recent reports have shown that expression of miR-206 is increased in muscles of mice and/or patients afflicted with DMD (9,20,26–28).

Recent reports have highlighted the potential role of miR-206 in the post-transcriptional downregulation of utrophin A in skeletal muscle cells (29–31). More specifically, it was shown in these various studies, that miR-206 can repress expression of reporter constructs containing the utrophin A 3′-UTR (untranslated region) leading to the notion that this regulatory loop can modulate endogenous utrophin A expression in muscle. These are important findings given that upregulation of utrophin A is currently envisaged as a potential therapeutic approach for altering the relentless progression of DMD. Several studies have clearly shown via transgenic technology (32–35) or pharmacological agents (36–38), the ability of increased utrophin A to functionally compensate for the lack of dystrophin in dystrophic fibers. In recent work, we identified another pathway also causing downregulation of utrophin A via post-transcriptional events (39). In this case, we showed using a variety of complementary approaches that K-homology splicing regulator protein (KSRP), a member of the AU-rich element binding protein (ARE-BP) family, binds directly to conserved AREs in the 3′-UTR of utrophin A mRNAs thereby increasing their destabilization and decay.

Given the recent description of these two post-transcriptional pathways implicating specific transacting factors, i.e. miR-206 and KSRP, which appear to both negatively regulate utrophin A expression, we sought in the current study to determine whether these two pathways act in concert to downregulate more markedly, perhaps even synergistically, utrophin A expression in skeletal muscle. Surprisingly, we discovered that miR-206 overexpression in cultured myogenic cells and dystrophic muscle fibers *in vivo* upregulates endogenous utrophin A levels. We show that this upregulation is caused by a novel pathway involving binding of miR-206 to conserved sites located in the 3′-UTR of KSRP, thus causing the subsequent inhibition of KSRP expression. This miR-206-mediated decrease in KSRP levels leads, in turn, to an increase in the expression of utrophin A transcript and protein due to a reduction in the activity of this destabilizing RNA-binding protein. Therefore, our study reveals that two apparent negative post-transcriptional pathways can, in fact, act distinctively as molecular switches causing repression or activation of utrophin A expression via its 3′-UTR according to the relative abundance of miR-206 versus KSRP, respectively.

## MATERIALS AND METHODS

### Cell culture, plasmids and transfection

Mouse C2C12 cells (American Type Culture Collection, Manassas, VA, USA) or mouse Neuro-2a (N2a) neuroblastoma cells (American Type Culture Collection, Manassas, VA, USA) were plated on 6-well culture dishes coated with Matrigel (BD Biosciences, Bedford, MA, USA) in Dulbecco’s modified Eagle’s medium (Invitrogen, Carlsbad, CA, USA) supplemented with 10% fetal bovine serum (Wisent, St-Bruno, QC, Canada), l-glutamine and penicillin/streptomycin and grown in a humidified chamber at 37°C with 5% CO2.

Transient transfections were performed using Lipofectamine 2000 (Invitrogen, Carlsbad, CA, USA) according to the manufacturer’s instructions. Cells at ∼40–50% confluency were incubated with the Lipofectamine2000/DNA mix for 4–5 h and harvested 15–24 h after transfection for analysis. A total of 50 pmol pre-miR-206 or pre-miR negative control #1 (Ambion, Austin, TX, USA) was transfected in both C2C12 and N2a cells. To inhibit miR-206 levels, miRIDIAN hairpin inhibitor mmu-miR-206 or negative control #1 (Thermo Fisher Scientific, Walthman, MA, USA) was transfected at 1 µg/ml, and the cells were harvested 48 h later.

The full-length 3′-UTR of the mouse utrophin A mRNA (∼2.1 kb) (39) and the full-length 3′-UTR of the mouse KSRP mRNA (∼1642 kb) were isolated by reverse transcriptase-polymerase chain reaction (RT-PCR) and subcloned downstream of the Firefly luciferase gene driven by the cytomegalovirus (CMV) promoter (pGL4). Cells were cotransfected with 250 ng Firefly luciferase and with 50 ng of Renilla luciferase reporter (phRGtk-luc) to control for transfection efficiency. For short hairpin RNA (sh-RNA) transfections, C2C12 cells (∼40% confluence) were plated in 6-well plates and transfected the following day in serum-free Dulbecco’s modified Eagle’s medium with KSRP-pRS-Sh-RNA (2 µg) or control pRS-Sh-RNA (2 µg), using Lipofectamine 2000 transfection reagent for 48 h.

Mouse KSRP complementary DNA was inserted into the EcoRI and XhoI sites of the pCMV-Myc vector. For overexpression studies, this construct or its empty control vector were transfected into N2a cells as described earlier in the text. Finally, mutations in the KSRP 3′-UTR were generated using the Quick Change Lightning site-directed mutagenesis kit (Strategene) and verified by DNA sequencing.

### Animals and electrotransfer of plasmid DNA

Six-week-old male mdx mice were maintained in the Animal Care and Veterinary Service of the University of Ottawa, under a constant 12 h light/dark cycle with food and water *ad libitum*. The experimental protocols were all approved by the University of Ottawa Institutional Animal Care and User Committee. Electrotransfer of pEGP-mmu-miR-206 or pEGP-mmu-miR-Null (Cell Biolabs Inc., San Diego, CA, USA) into tibialis anterior (TA) muscles was performed as described elsewhere (39,40) while the animals were under anesthesia. Ten days after electroporation, the mice were euthanized and muscles were frozen in liquid nitrogen. All muscle samples were stored at −80°C for subsequent analyses.

### Protein extraction and immunoblot analyses

Proteins were extracted from powdered muscles by homogenization at 4°C in radioimmunoprecipitation assay (RIPA) buffer (Sigma-Aldrich). Homogenates were centrifuged at 12 000*g* for 12 min at 4°C, and supernatants were collected and stored in aliquots at −80°C. Protein concentration was measured using the Bio-Rad DC Protein Assay kit. For western blotting, 50 µg of proteins were subjected to SDS–PAGE and transferred to nitrocellulose membranes. Gel loading was systematically checked by coomassie blue and Ponceau S staining. Blots were incubated overnight at 4°C with primary antibodies against utrophin A (Novocastra NCL-DRP2, Newcastle upon Tyne, UK), KSRP (Bethyl Laboratories), glyceraldehyde-3-phosphate dehydrogenase (GAPDH; Advanced Immunochemical, Long Beach, CA, USA), mouse anti-ß-actin (Santa Cruz Biotechnology) and mouse anti-Pax 3 (Developmental Studies Hybridoma Bank). Murine hybridomas producing a mouse monoclonal antibody to KSRP (Ab5) were provided by Dr Douglas L Black (Department of Microbiology, Immunology and Molecular Genetics, University of California, Los Angeles, CA, USA) (41). Incubation with corresponding horseradish peroxidase-conjugated rabbit anti-mouse antibodies (KPL, Kirkegaard Perry laboratories), rabbit anti-goat antibodies (Jackson ImmunoResearch) or horseradish peroxidase-conjugated-protein A (ZYMED) was performed for chemiluminescent detection of proteins (ECL; PerkinElmer). The films were scanned and quantified using NIH Image version 1.63. 

### KSRP immunoprecipitation

C2C12 cells were lysed with RIPA buffer and incubated with a rabbit polyclonal KSRP antibody overnight at 4°C as recently described (39). Complexes were immunoprecipitated using protein G Sepharose (Sigma Aldrich, St Louis, MO, USA) for 3 h at 4°C and then washed with RIPA buffer supplemented with protease and phosphatase inhibitors, and analyzed by resolving 10% SDS–PAGE and immunoblotting with KSRP antibodies. 

### RNA extraction and RT-PCR

Total RNA was extracted from TA muscle and C2C12 or N2a cells using TRIzol reagent (Invitrogen) as recommended by the manufacturer. TRIzol-extracted RNA was treated for 1 h with DNAse I (Invitrogen) to eliminate possible DNA contamination. Reverse transcription (RT) was carried out using an RT reaction mixture containing 5 mM MgCl_2_, 1× PCR buffer, 1 mM dNTP, 1 U/µl RNase inhibitor, 5 U/µl Moloney murine leukemia virus reverse transcriptase and 2.5 µM random hexamers (Applied Biosystems, CA, USA).

Real-time quantitative PCR was performed on an MX3005p real-time PCR system (Stratagene, La Jolla, CA, USA) using QuantiTect SYBR Green PCR kit (QIAGEN, Valencia, CA, USA). For these experiments, amplification of 18S ribosomal subunit, GAPDH, utrophin A and KSRP was performed in triplicate with the following primer sequences: utrophin, forward 5′-ATCTTGTCGGGCTTTCCAC-3′ and reverse 5′-ATCCAAAGGCTTTCCCAGAT-3′, KSRP forward 5′TTATCGGGGACCCATACAAA-3′ and reverse 5′-ACTCCGGCCAATGACTACAC-3′, 18S ribosomal forward 5′-CGCCGCTAGAGGTGAAATC-3′ and reverse 5′-CCAGTCGGCATCGTTTATGG-3′ and GAPDH forward 5′-GGGTGTGAACCACGAGAAAT-3′and reverse 5′-CCTTCCACAATGCCAAAGTT-3′. The expression levels were quantified and normalized to 18S ribosomal RNA and/or GAPDH.

### MicroRNAs detection

For detection of endogenous miRNA levels, miScript reverse transcription kit (Qiagen) was used for retrotranscribed according to manufacturers’ instructions. Quantitative qRT-PCR and PCR reactions were performed using the miScript SYBR green PCR kit (Qiagen) with primers designed by the manufacturer.

### Immunofluorescence

Cells were initially fixed with 4% paraformaldehyde in 1× phosphate-buffered saline (PBS) for 5 min at room temperature and immunostained using antibodies against utrophin A or KSRP. Appropriate secondary antibodies were coupled to Alexa 594 (Molecular Probes, Eugene, OR, USA). Immunostained cells were visualized with a Z.1 AxioImager upright microscope (Carl Zeiss, Canada). Images were captured through an EC PLAN-NEOFLUAR 20X/0.5 M27 objective and an AXIOCAM HRM R 2.0 CCD camera. Muscle crosssections (10 µm) were cut in a microtome at −20°C and stained with utrophin antibodies along with Alexa 488 conjugated α-bungarotoxin to label neuromuscular junctions (Jackson ImmunoResearch, West Grove, PA, USA). Immunofluorescence experiments were performed simultaneously on TA muscle from mdx mice electroporated with pEGP-mmu-miR-Null or pEGP-mmu-miR-206, placed on the same slides. Quantification of the fluorescence intensity was performed using ImageJ. 

### Luciferase assays

C2C12 cells were homogenized (1:10 dilution, w/v) in reporter lysis buffer (Dual Luciferase Assay System, Promega, Madison, WI, USA), subsequently frozen in liquid nitrogen and thawed and refrozen three times at 37°C. The activity of Firefly and Renilla luciferase was determined using the Dual Luciferase Assay kit. To correct for variations in transfection efficiency, Firefly luciferase activity was normalized to Renilla luciferase activity.

### RNA Immunoprecipitation

RNA immunoprecipitations (RIP) were performed as recently described (39). C2C12 cells were crosslinked with 1% formaldehyde in PBS for 10 min at room temperature. The reaction was stopped with a wash in ice-cold PBS. Equal amounts of whole-cell extracts were immunoprecipitated with Protein A agarose-bound rabbit anti-KSRP antibody (Bethyl Laboratories) beads (Sigma) or rabbit IgG (Santa Cruz Biotechnology, Santa Cruz, CA, USA) as control. The beads were washed with modified RIPA buffer supplemented with protease and phosphatase inhibitors (Roche) and heated to 70°C for 1 h to reverse crosslinking. RNA was extracted using TRIzol reagent (Invitrogen). Real-time quantitative PCR was performed as described earlier in the text.

### Statistics

Various statistical tests including paired and unpaired *t-*tests as well as one-way analysis of variance followed by Bonferroni test were used to determine whether specific group mean differences were significant. Each test performed is specified in the figure legends. The minimum α-level of significance was set at 0.05. Data are presented as means ± SEM throughout.

## RESULTS

### MicroRNA-206 enhances endogenous utrophin A expression

Recently, we reported that utrophin A mRNA is a direct target of KSRP in cultured myogenic cells as well as in skeletal muscle fibers of mdx mice, causing destabilization of existing transcripts (39). On the other hand, independent reports using cultured cells have shown that miR-206 targets the 3′-UTR of utrophin A and reduces expression of reporter constructs via one conserved seed sequence (29,30). Together, these studies suggest the existence of parallel post-transcriptional pathways converging onto the utrophin A 3′-UTR that negatively regulate utrophin A expression. Given these observations, we therefore initially wondered whether the regulation of utrophin A by miR-206 could also influence the endogenous expression of utrophin A.

**Figure 1.**
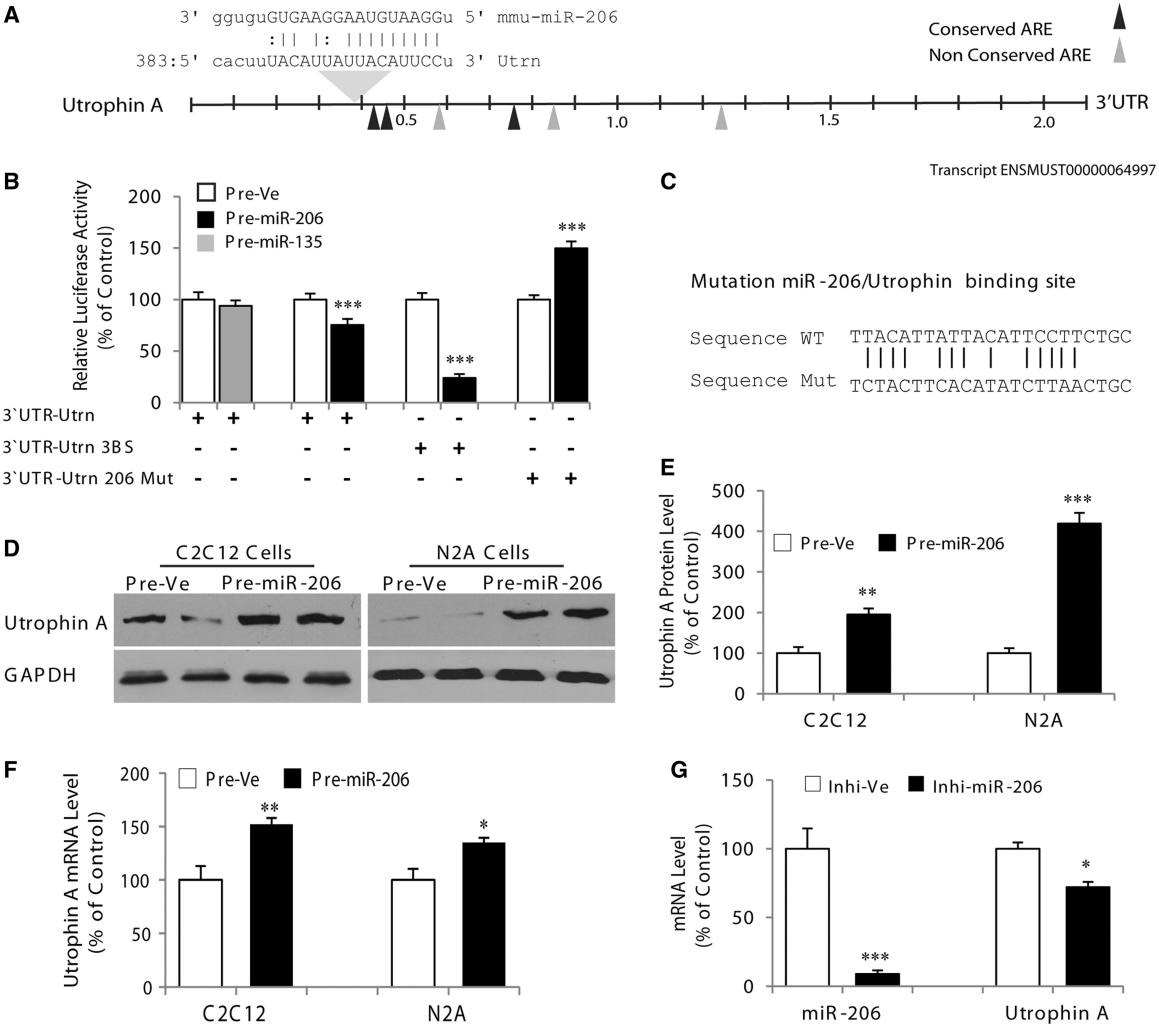
MicroRNA-206 enhances endogenous utrophin A expression. (**A**) Schematic representation of the utrophin A 3′-UTR showing the presence of a binding site of miR-206. (**B**) C2C12 myoblasts were cotransfected with reporter constructs containing the utrophin A full-length 3′-UTR, utrophin A full-length 3′-UTR with miR-206 seed sequence mutated or utrophin A full-length 3′-UTR with three binding sites (3BS) of miR-206, together with pre-miR-206 or pre-Ve. Pre-miR-135, a miRNA not predicted to target KSRP, was also used as control. (**C**) Shows the conserved binding site of miR-206 that was mutated within the utrophin A full-length 3′-UTR. (**D**) Representative immunoblots of utrophin A protein levels after pre-miR-206 or pre-Ve transfection in C2C12 cells and N2a cells. For the western blots, GAPDH was used to ensure equal loading. (**E**) Represents quantification of utrophin A protein levels in C2C12 cells and N2a cells transfected with pre-miR-206 or pre-Ve. (**F**) RT-qPCR analyses of utrophin A mRNA expression in C2C12 cells and N2a cells transfected with pre-miR-206 or pre-Ve. Note the increase in utrophin A protein and mRNA levels on pre-miR-206 overexpression. (**G**) Shows the results of RT-qPCR analyses for utrophin A mRNA in C2C12 cells transfected with the miR-206 inhibitor (150 nM). Values are means ± standard error (SE) (*n* = 3/group in triplicate). *P < 0.05; **P < 0.01; ***P < 0.001; relative to corresponding control (Figure 1B, E–G: unpaired *t*-tests).

To address this, we first cotransfected into myoblasts the synthetic pre-miR-206 with a luciferase reporter construct containing the utrophin A 3′-UTR. Consistent with previous reports that have used a similar approach (29,30,42), we observed a modest but statistically significant decrease (*P* < 0.001) in luciferase activity in C2C12 cells overexpressing miR-206 (Figure 1A and B). Mutation of the miR-206 site in the utrophin A 3′-UTR (Figure 1C) led to an increased expression of luciferase (*P* < 0.001). Moreover, previous work also showed that artificially engineering 3 miR-206 binding sites into the utrophin A 3′-UTR results in efficient and greater downregulation by miR-206 (30). In our hands, we also observed that the presence of 3 miR-206 binding sites into an engineered reporter construct containing the utrophin A 3′-UTR caused a greater reduction (*P* < 0.001) in luciferase activity on overexpression of miR-206. Because miR-1 shares the same seed sequence as miR-206 and constitutes an alternative to the regulation of utrophin A, we also tested whether miR-1 could repress utrophin A expression. As expected, and similar to what was observed with miR-206, miR-1 is able to repress expression of a luciferase reporter containing the 3′-UTR of utrophin A (Supplementary Figure S1).

To examine whether this mechanism translates into a physiological regulation at the level of endogenous utrophin A, we used western blot and RT-qPCR to assess the expression of utrophin A protein and mRNA, respectively, in two different cell lines, namely, C2C12 and N2a cells. It should be noted that N2a cells do not endogenously express miR-206. Surprisingly, on overexpression of miR-206, utrophin A protein levels were highly increased (*P* < 0.01) in both cell types (Figure 1D and E). Furthermore, quantitative analysis revealed that utrophin A transcripts were augmented by ∼ 50% (*P* < 0.05) in C2C12 and N2a cells overexpressing miR-206 (Figure 1F). In a reciprocal experiment, inhibition of miR-206 resulted in a significant decrease (*P* < 0.05) in the levels of utrophin A mRNA (Figure 1G).

### MicroRNA-206 targets the KSRP 3′-UTR

**Figure 2.**
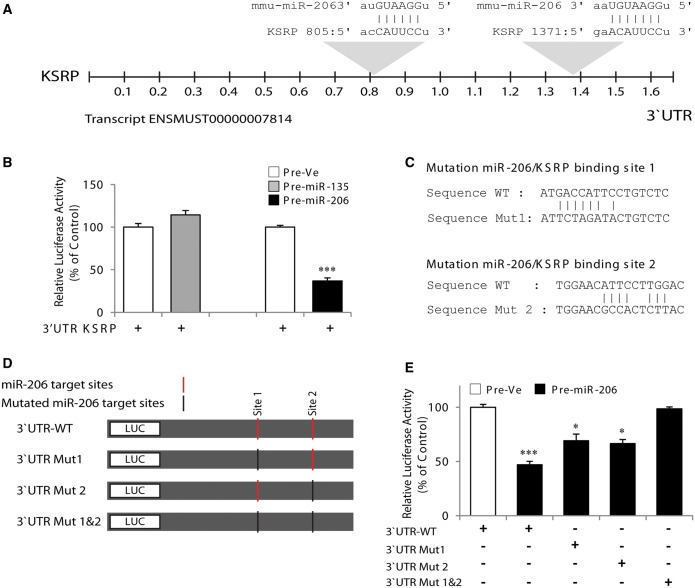
MicroRNA-206 targets the KSRP 3′-UTR. (**A**) Schematic representation of the mouse KSRP 3′-UTR showing the presence of binding sites for miR-206. (**B**) C2C12 cells were cotransfected with a reporter construct containing the mouse 3′-UTR of KSRP, together with pre-miR-206 or pre-Ve. Pre-miR-135, a miRNA not predicted to target KSRP, was also used as control. (**C**) Base pairings of miR-206 with the 3′-UTR KSRP showing sites of mutations. (**D**) Schematic representation of reporter constructs containing the wild-type 3′-UTR of KSRP or mutated versions. Red vertical bars represent miR-206 target sites; black vertical bars represent mutated miR-206 target sites. (**E**) Shows luciferase activity observed with the different 3′-UTR constructs of KSRP on overexpression of pre-miR-206 in C2C12 cells. Values are means ± SE (*n* = 3/group in triplicate). *P < 0.05; ***P < 0.001 relative to corresponding control (Figure 2B: unpaired *t*-test; Figure 2E; One-way ANOVA and Bonferroni as post hoc test).

Based on the results presented earlier in the text, we surmised that miR-206 might directly and preferentially regulate expression of KSRP, which we have shown recently to cause destabilization of utrophin A transcripts at the post-transcriptional level (39). Such a mechanism could explain why overexpression of miR-206 results in an increase in endogenous utrophin A expression. To test this possibility, we first examined the 3′-UTR of the mouse KSRP transcript, which is 1642 nt in length. Its analysis using a miRNA Target Prediction algorithm (TargetScan and MicroRNA), revealed the presence of potential target sites for miR-206 (Figure 2A). Moreover, our analysis uncovered that these motifs are conserved across human, mouse and rat.

To determine whether miR-206 regulates KSRP expression, we first inserted the mouse 3′-UTR of KSRP in a luciferase reporter construct. Overexpression of miR-206 in myoblasts transfected with this reporter construct caused a large decrease (*P* < 0.001) in luciferase activity as compared with that observed in control cells and cells overexpressing miR-135; a miRNA not predicted to target KSRP (Figure 2B). To evaluate the functional contribution of each miR-206 sites present in the KSRP 3′-UTR, we mutated these sites in the mouse full-length 3′-UTR of KSRP (Figure 2C and D). Transfection of myoblasts with reporter constructs containing the full-length KSRP 3′-UTR with individual mutations of miR-206 sites significantly attenuated (*P* < 0.001) the repressive effect of pre-miR-206 (Figure 2E). Moreover, mutation of both target sites completely abrogated (*P* > 0.05) the repressive effect of miR-206 (Figure 2E).

**Figure 3.**
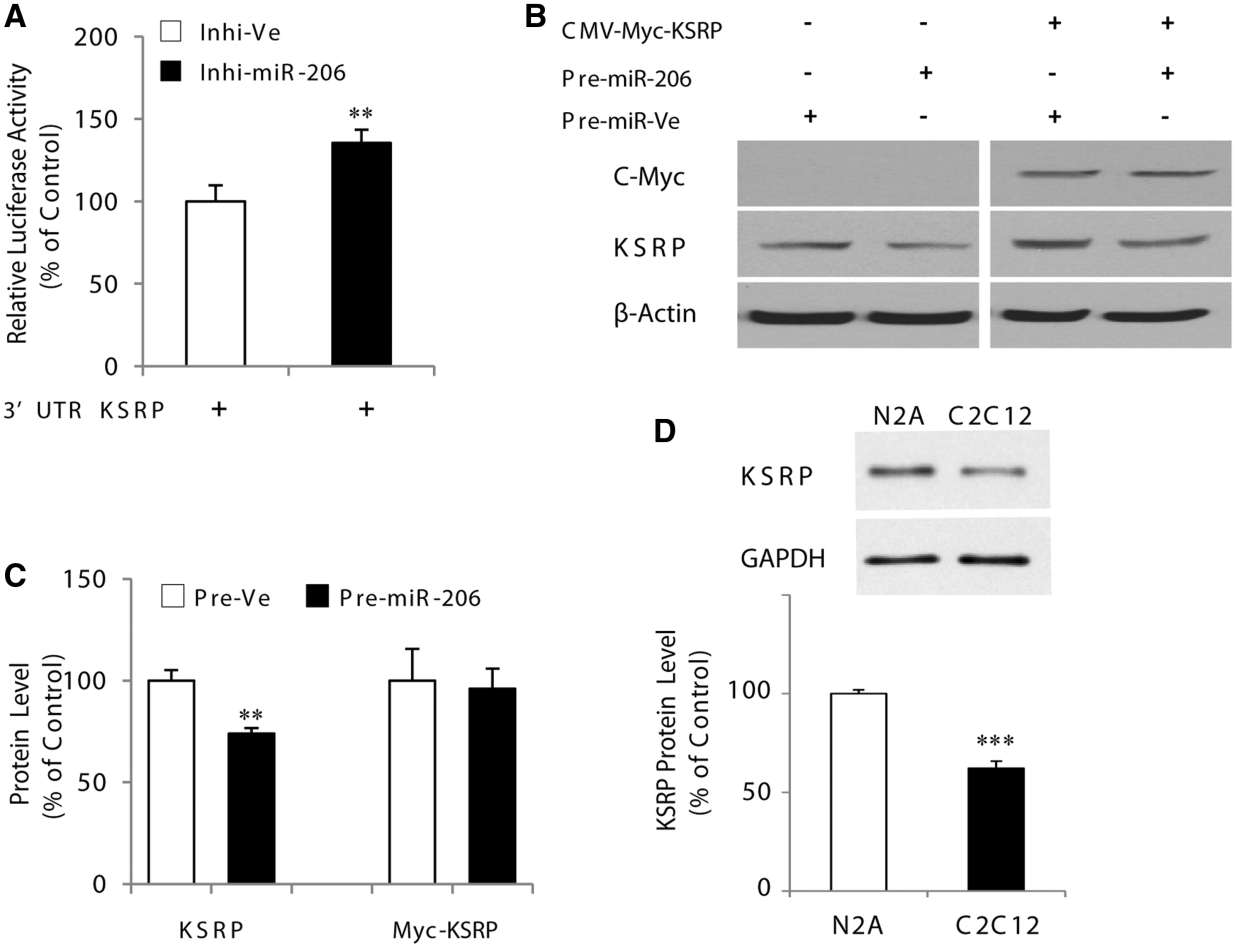
MicroRNA-206 specifically regulates KSRP expression via its 3′-UTR. (**A**) Represents the activity of the luciferase reporter construct containing the mouse KSRP 3′-UTR in C2C12 cells transfected with miR-206 inhibitor (100 nM) or negative control. (**B**) and (**C**) show representative western blots and their quantification, respectively, for KSRP and C-Myc with β-actin as a control. (**D**) Representative western blots and their quantification of KSRP protein levels in N2a and C2C12 cells. Values are means ± SE (*n* = 3/group in triplicate for luciferase experiments). **P < 0.01; ***P < 0.001; relative to corresponding control (Figure 3A–D: unpaired *t*-test).

To confirm this regulatory mechanism, we performed additional and complementary experiments using a specific inhibitor of miR-206. In these experiments, we first verified that expression of the mature miR-206 was repressed with this specific inhibitor (Figure 1G). Transfection of the KSRP 3′-UTR-reporter construct with the miR-206-specific inhibitor caused a significant increase of close to 50% (*P* < 0.01) in luciferase activity as compared with control cells (Figure 3A). Additionally, we examined whether the KSRP open-reading frame is resistant to miR-206 expression due to the absence of the 3′-UTR. To this end, we cotransfected a KSRP open-reading frame construct driven by the cytomegalovirus promoter and fused to a Myc-epitope tag (pCMV-Myc-KSRP), along with pre-miR-206. As demonstrated in Figure 3B, the level of Myc-tagged KSRP, was not affected (*P* > 0.05) by miR-206 overexpression. Importantly, however, the endogenous levels of KSRP protein were significantly decreased (*P* < 0.01) under these conditions (Figure 3B and C). Taken together, these results demonstrate that the two miR-206 binding sites located in the mouse KSRP 3′-UTR are functional and can cause repression of KSRP expression. In addition, we performed western blots to determine the levels of KSRP expression in C2C12 versus N2a cells and observed that N2a cells express significantly (*P* < 0.05) more KSRP. Such a finding is in fact consistent with the greater impact of miR-206 on endogenous utrophin A protein expression seen in N2a cells (see Figure 1E).

### MicroRNA-206 regulates expression of endogenous KSRP

**Figure 4.**
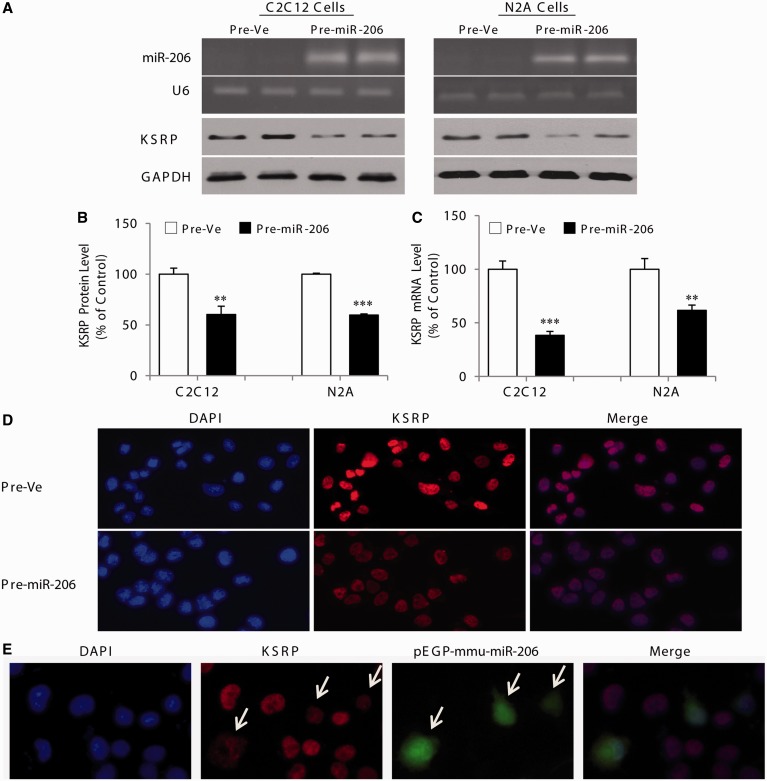
MicroRNA-206 regulates expression of endogenous KSRP. (**A**) Top two panels: representative ethidium bromide-stained agarose gel showing mature miR-206 RT-PCR product amplified from C2C12 and N2a cells transfected with pre-miR-206. The U6 was used as control for loading. Bottom two panels: representative immunoblots of KSRP protein levels after pre-miR-206 transfection in C2C12 and N2a cells. (**B**) and (**C**) western blot quantification and qRT-PCR, respectively, for KSRP in C2C12 and N2a cells transfected with a pre-miR-206 or pre-Ve. (**D**) Shows immunofluorescence experiments for KSRP in control N2a cells (pre-Ve) or cells transfected with pre-miR-206. (**E**) Immunofluorescence for KSRP in N2a cells transfected with a mmu-miR-206 construct coupled with GFP (pEGP-mmu-miR-206).The reduction in KSRP levels was only seen in GFP-positive cells (arrows). As a control, no staining was observed in experiments with no primary antibody. Values are means ± SE (*n* = 3/group in triplicate). **P < 0.01; ***P < 0.001; relative to corresponding control (Figure 4B and C: unpaired *t*-test).

To further determine whether miR-206 affects endogenous KSRP expression, we transfected C2C12 and N2a cells with the synthetic miR-206 precursor RNA (pre-miR-206). Under these conditions, overexpression of miR-206 induced a ∼40% (*P* < 0.01) decrease in KSRP protein levels in both cell types (Figure 4A and B). Similar to the decrease seen in KSRP protein, levels of KSRP transcript were also significantly repressed (*P* < 0.01) in both cell types following overexpression of miR-206 (Figure 4C). In addition, we performed immunofluorescence experiments to further examine the effects of miR-206 overexpression on KSRP levels in C2C12 myoblasts. As shown in Figure 4D, the staining intensity corresponding to KSRP expression was markedly reduced on overexpression of pre-miR-206 as compared with a respective control. In parallel experiments, overexpression of miR-206 was also achieved in selected cells via a mmu-miR-206 construct coupled with green fluorescent protein (GFP) (pEGP-mmu-miR-206). As illustrated in Figure 4E, we observed marked reduction in KSRP levels but only in GFP-positive cells as expected (arrows). In these experiments, we observed a decrease in KSRP expression in a higher proportion of cells treated with pre-miR-206 (Figure 4D) as compared with cells transfected with pEGP-mmu-miR-206 (Figure 4E). Also, in these experiments, a no-primary antibody control showed no fluorescence indicating the specificity of the KSRP signal seen under these conditions (not shown).

**Figure 5.**
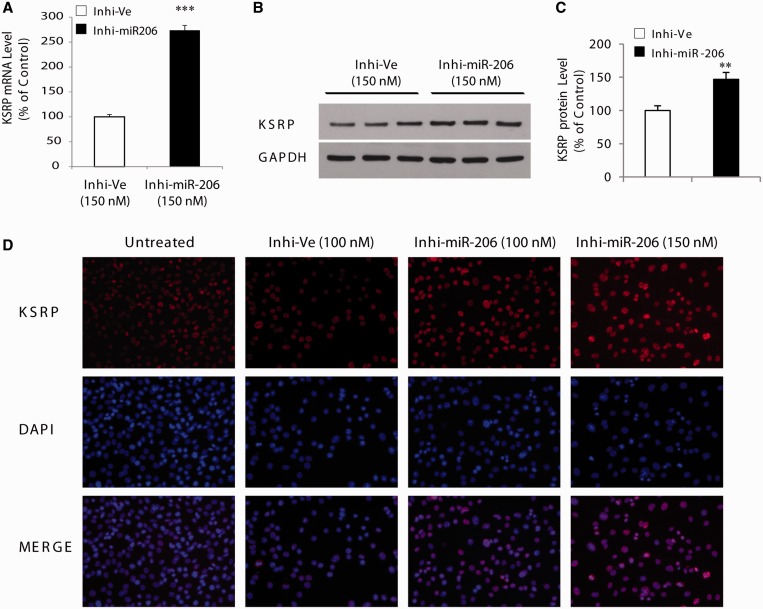
Inhibition of miR-206 causes derepression of KSRP. (**A**) Inhibition of miR-206 promotes increases in KSRP expression in C2C12 cells. Real-time qPCR analysis revealed that the endogenous mRNA levels of KSRP were increased with the miR-206 inhibitor at 150 nM. (**B**) Shows western blot analysis of KSRP with GAPDH as control. (**C**) Represents quantification of KSRP protein levels in C2C12 cells transfected with the miR-206 inhibitor. (**D**) Shows immunofluorescence experiments for KSRP in control N2a cells transfected with miR-206 inhibitor at 100 nM and 150 nM or negative control at 100 nM. Note the increase in KSRP in the presence of the inhibitor. As a control, no staining was observed in experiments with no primary antibody. Values are means ± SE (*n* = 3/group in triplicate). **P < 0.01; ***P < 0.001; relative to corresponding control (Figure 5A: one-way ANOVA and Bonferroni as post hoc test; Figure 5C: unpaired *t*-test).

We also performed additional experiments using the specific inhibitor of miR-206. In perfect agreement with the impact of inhibiting miR-206 on expression of the reporter construct containing the 3′-UTR of KSRP (Figure 3B), the endogenous mRNA level of KSRP was dose-dependently increased (*P* < 0.001) in the presence of the miR-206-specific inhibitor (Figure 5A). Importantly, the highest concentration of miR-206-specific inhibitor transfected into C2C12 myoblasts caused a ∼50% increase (*P* < 0.01) in KSRP protein expression (Figure 5B and C). The increased expression of KSRP in C2C12 myoblasts treated with the specific inhibitor of miR-206 was further confirmed by immunofluorescence staining in which a clear increase in KSRP levels was noted following treatment with the inhibitor (Figure 5D). Taken together, these results show that mir-206 regulates the endogenous expression of both KSRP mRNA and protein in myogenic cells.

### Expression of KSRP and miR-206 is inversely correlated during skeletal muscle cell differentiation

**Figure 6.**
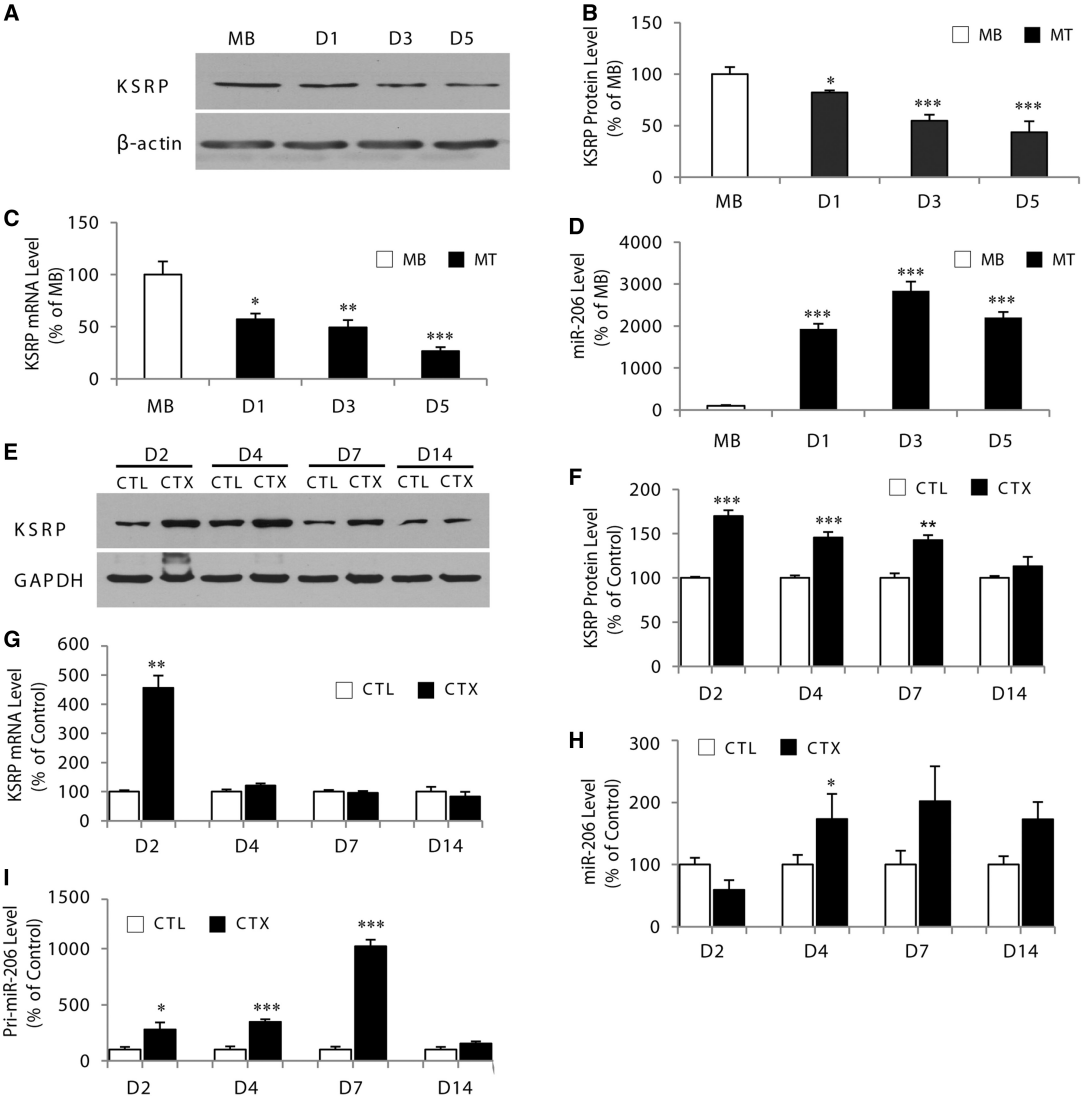
Expression of KSRP and miR-206 is inversely correlated during skeletal muscle cell differentiation and after CTX-induced degeneration/regeneration. (**A**) Representative western blot showing KSRP level in myoblasts (MB) and myotubes at day 1, 3 and 5 of differentiation. (**B**) KSRP levels were quantified and are expressed as a percent of the levels seen in myoblasts. The ß-actin served as loading control. (**C**) RT-qPCR of KSRP mRNA levels during differentiation of C2C12 myoblasts. (**D**) RT-qPCR analysis of miR-206 expression normalized to U6 levels in C2C12 myoblasts and myotubes at day 1, 3 and 5. (**E**) Representative western blots showing KSRP levels in TA muscles from mdx mice injected with cardiotoxin (CTX) or saline (as a control: CTL) 2, 4, 7 and 14 days after injection. (**F**) Western blot analysis of KSRP protein levels after CTX-induced muscle degeneration and regeneration. (**G**) Represents quantification of KSRP mRNA levels in TA muscles from mdx mice after CTX-induced injury. (**H**) and (**I**) RT-qPCR revealed an increase in miR-206 and pri-miR-206 levels during CTX-induced injury. Values are means ± SE (*n* = 4/group). **P < 0.01; ***P < 0.001; relative to corresponding control (Figure 6B: unpaired *t*-test; Figure 6B–D: unpaired one-way ANOVA and Bonferroni as post hoc test; Figure 6F–I: paired one-way ANOVA and Bonferroni as post hoc test). At D14 in Figure 6H, *P* = 0.059.

To complement these studies, we also determine the expression of KSRP and miR-206 during myogenic differentiation. Stimulation of C2C12 cells differentiation by serum withdrawal caused a marked decrease in the expression of KSRP protein (Figure 6A and B). The decline in KSRP protein level was progressive and reached by day 5 of differentiation, ∼50% (*P* < 0.001) of the levels seen in myoblasts (Figure 6B). In addition, levels of KSRP mRNA were also significantly decreased (*P* < 0.001) in myotubes as compared with myoblasts (Figure 6C). These results show that expression of KSRP is regulated during myogenic differentiation. By contrast, and in agreement with previous work (22,25,43), expression of miR-206 dramatically increased (*P* < 0.001) on induction of differentiation (Figure 6D). Consistent with our model of regulation, levels of utrophin A were also increased during differentiation, thereby mirroring the decrease in KSRP expression (Supplementary Figure S2).

In separate experiments, we examined whether this inverse correlation between KSRP and miR-206 levels also occurs in differentiating muscle fibers *in vivo*. Given the relevance of miR-206 and KSRP to the dystrophic phenotype (18,39), we triggered muscle fiber degeneration/regeneration in mdx mice, a widely used model of DMD, by injecting cardiotoxin (CTX) in the TA muscle. This model is known to recapitulate the molecular and cellular events seen in differentiating myogenic cells in culture. As presented in Figure 6E, F and G, KSRP protein and mRNA levels were upregulated (*P* < 0.001) 2 days after CTX injury. The KSRP protein levels subsequently decreased steadily between days 4–14 after injury (Figure 6E and F), whereas KSRP transcripts returned to control levels more rapidly (Figure 6G). As previously described (18,44), miR-206 levels remained initially unchanged at day 2 following CTX-induced injury, but then increased (*P* < 0.05) and remained relatively stable afterward (Figure 6H). This result indicates that the ∼450% increase in KSRP mRNA seen at early stages of regeneration (2 days) does not occur via miR-206 and further suggests, therefore, that additional mechanisms acting at the transcriptional level and/or at the level of transcript stability with the contribution of additional transacting factor(s), are involved in this large KSRP mRNA increase. In addition, expression of KSRP protein does not match the expression pattern of KSRP mRNA (Figure 6F and G), which could indicate that at later stages of regeneration (4–14 days) miR-206 can act at the translational level on KSRP transcripts. The pattern of miR-206 expression during regeneration is also consistent with the increase seen in the abundance of primary miRNA-206 (pri-miR-206) after CTX-induced injury (Figure 6I) (45). Taken together, it appears therefore that expression of KSRP and miR-206 tends to be inversely correlated during myogenic differentiation of cultured cells and during muscle fiber regeneration *in vivo*. These findings emphasize under physiological conditions that miR-206 exerts a strong regulatory effect on KSRP expression.

### Competition of the miR-206 and KSRP pathways on the regulation of utrophin A

**Figure 7.**
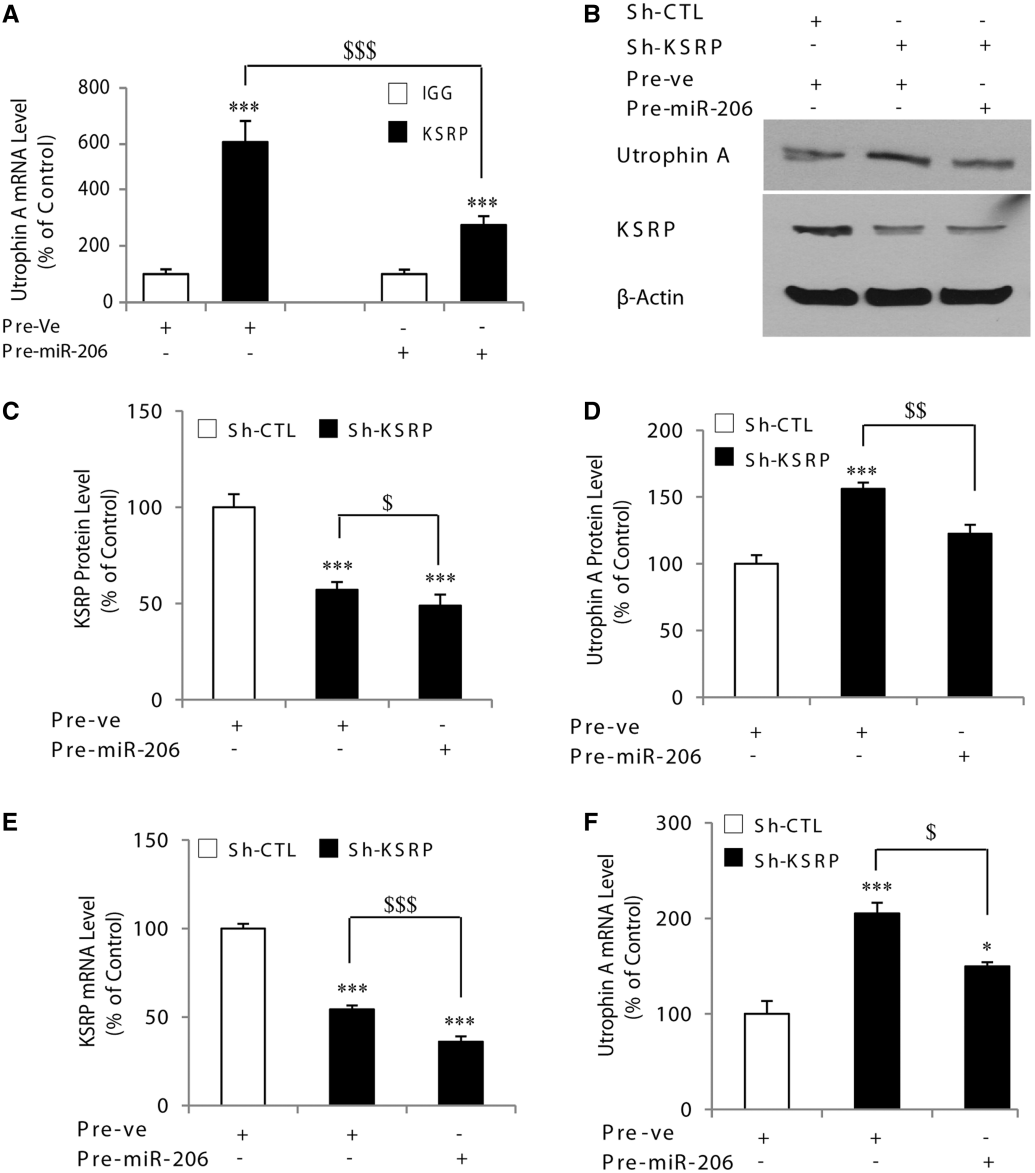
Competition of the miR-206 and KSRP pathways on the regulation of utrophin A. (**A**) Represents the results of RIP assays in C2C12 cells transfected with either negative control or pre-miR-206. Samples were immunoprecipitated with either IgG as control or with an antibody against KSRP. GAPDH was used as a control. Real-time qPCR analysis revealed a decreased amount of utrophin A mRNA interacting with KSRP on miR-206 overexpression. (**B**) Representative western blots of utrophin A and KSRP in C2C12 cells transfected with pre-miR-206 or negative control and with sh-RNA against KSRP. The ß-actin served as a loading control. (**C** and **D**) Represents quantification of KSRP and utrophin A protein levels in C2C12 cells transfected with pre-miR-206 or negative control and with sh-RNA against KSRP. (**E** and **F**) Show the results of RT-qPCR analyses for KSRP and utrophin A mRNA, respectively, in C2C12 cells transfected with pre-miR-206 or negative control and with sh-RNA against KSRP. Levels of KSRP and utrophin A mRNA are standardized to 18S mRNA. Values are means ± SE (*n* = 4/group). *P < 0.05; **P < 0.01, ***P < 0.001; relative to corresponding control (Figure 7A, C–F: one-way ANOVA and Bonferroni as post hoc test). $ < 0.05; $$ < 0.01; $$$ < 0.001.

Given the data shown earlier in the text demonstrating the impact of miR-206 on KSRP expression, we decided to next assess whether the observed increase in endogenous utrophin A expression caused by miR-206 occurs via the regulation of KSRP in cells. To examine this, we initially determined whether the pattern of interaction between KSRP and utrophin A mRNA was altered by overexpression of miR-206. The RIP assays with C2C12 cells transfected with pre-miR-206 or pre-Ve were performed using a KSRP antibody. In agreement with our endogenous expression data, the extent of interaction between KSRP and utrophin A mRNA was significantly reduced (*P* < 0.01) in cells transfected with pre-miR-206 (Figure 7A), which, therefore, can account for the increased utrophin A expression seen under these conditions. Additionally, these results demonstrate that although miR-206 can target the utrophin A 3′-UTR when overexpressed in a reporter construct, it appears that in a physiological setting, it preferentially targets and regulates the relative abundance of KSRP which, in turn, affects endogenous utrophin A levels via recruitment of the mRNA degradation machinery to its 3′-UTR. Therefore, competing post-transcriptional pathways converge on the utrophin A 3′-UTR to regulate its expression, and this regulatory switch likely depends on the relative abundance of each component, namely, KSRP, miR-206 and the utrophin A 3′-UTR itself.

To investigate this model, we overexpressed miR-206 in myoblasts with reduced KSRP levels achieved by knockdown with a short hairpin RNA. As shown in Figure 7B–F, the knockdown of KSRP concomitant with overexpression of miR-206, induced a significant decrease (*P* < 0.01) of both utrophin A protein and mRNA, compared with cells with reduced KSRP levels only. Thus, in the presence of reduced levels of KSRP, miR-206 can exert a direct negative regulation on endogenous utrophin A expression. Collectively, these results demonstrate the dual competition between (i) the inhibitory effect of miR-206 on the 3′-UTR of utrophin A and (ii) the opposing positive effects of miR-206 on endogenous expression of utrophin A through repression of KSRP.

### MicroRNA-206 represses KSRP expression causing upregulation of utrophin A in mdx mouse skeletal muscle

**Figure 8.**
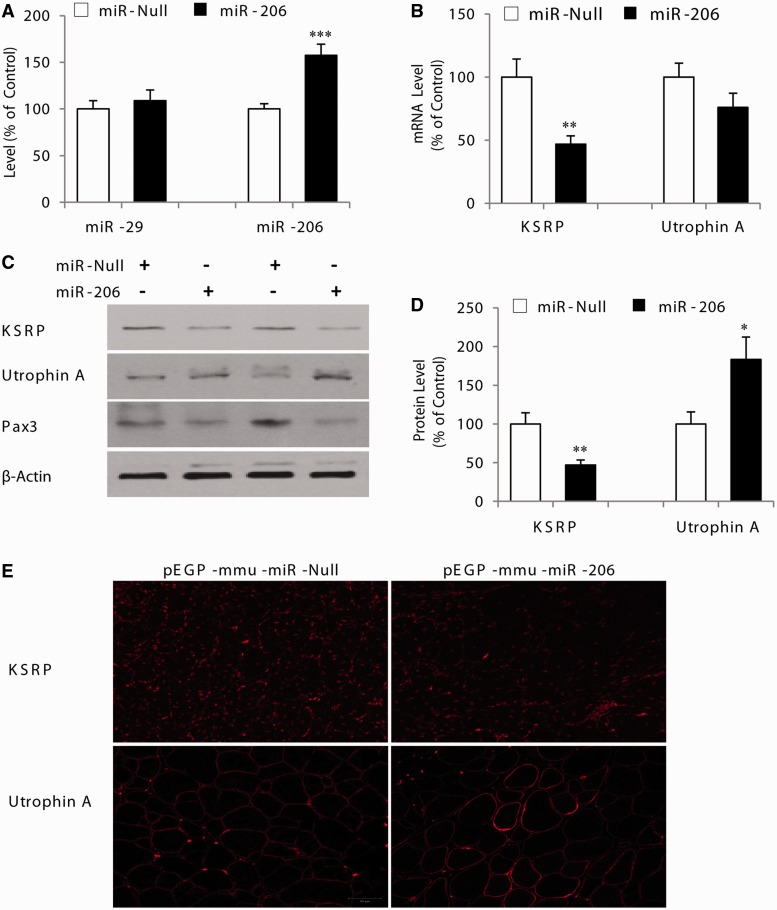
MicroRNA-206 represses KSRP expression causing upregulation of utrophin A in mdx mouse muscle fibers. The TA muscles from mdx mice were electroporated with pEGP-mmu-miR-206 or the corresponding control (pEGP-mmu-miR-Null in the contralateral TA muscle) and 10 days later, both TA muscles were excised and analyzed. (**A**) Shows the results of RT-qPCR analyses for miR-206 level in TA muscles electroporated with pEGP-mmu-miR-206 or pEGP-mmu-miR-Null. MiR-29C was used as a control. (**B**) Shows the results of RT-qPCR analyses for utrophin A and KSRP mRNAs in TA muscles electroporated with pEGP-mmu-miR-206 or pEGP-mmu-Null. (**C**) Shows representative western blot analysis of KSRP, utrophin A and Pax3 with β-actin as control for two different samples for each treatment. (**D**) Represents quantification of utrophin A and KSRP protein expression in TA muscles electroporated with miR-206 vector or corresponding control. (**E**) Are representative examples of muscle cross sections stained with an antibody against KSRP and utrophin A from mdx mouse muscle electroportated with pEGP-mmu-miR-206 or pEGP-mmu-miR-Null. As a control, no staining was observed in experiments with no primary antibody. Values are means ± SE (*n* = 6–8). *P < 0.05; **P < 0.01; ***P < 0.001; relative to corresponding control (Figure 1A, B and D: paired *t*-test).

To validate *in vivo* the post-transcriptional regulation of KSRP by miR-206 observed in cultured myogenic and neuronal cells, we examined the effects of miR-206 overexpression achieved by electroporation of mdx mouse muscle. Injection/electroporation of pEGP-mmu-miR-206 or pEGP-mmu-miR-Null was carried out in TA muscles, and the level of transduction was assessed by immunostaining for GFP coexpressed by both vectors. One TA muscle was used as the experimental side, whereas the contralateral TA was used for electrotransfer of the control vector. Ten days after injection/electroporation of pEGP-mmu-miR-206, miR-206 was selectively overexpressed in these dystrophic TA muscles (*P* < 0.001) compared with contralateral TA muscles (Figure 8A). By comparison, the levels of another miRNA, miR-29C, used in this case as a control for specificity, did not vary (*P* > 0.05) following overexpression miR-206. As seen with cultured myogenic cells, KSRP mRNA and protein levels were strongly decreased (*P* < 0.01) in response to the induction of miR-206 expression in dystrophic muscle (Figure 8B–D). In addition, a known target of miR-206, Pax3, was also downregulated on miR-206 overexpression in dystrophic muscle fibers (Figure 8C).

We also examined the impact of the post-transcriptional repression of KSRP by miR-206 in dystrophic muscle fibers. The changes in the abundance of KSRP were mirrored by a significant increase (*P* < 0.01) in utrophin A protein expression (Figure 8C and D). Moreover, the distinctive punctate immunofluorescence staining of KSRP in muscle was clearly decreased in dystrophic TA muscle injected with pEGP-mmu-miR-206 compared with its respective control (Figure 8E). Importantly, levels of utrophin A at the sarcolemma were increased following overexpression of miR-206 and the accompanied decrease in KSRP abundance (Figure 8E). Collectively, these results indicate that miR-206 controls indirectly and through KSRP, the relative abundance of utrophin A in dystrophic muscle fibers *in vivo*.

## DISCUSSION

Recent work has shown the existence of two post-transcriptional pathways that appear to cause repression of utrophin A expression in skeletal muscle. Several reports (29–31) have demonstrated that miR-206 can cause repression of reporter constructs containing the utrophin A 3′-UTR through a functional binding site. Moreover, we have shown recently that KSRP, a RNA-binding protein known to destabilize target transcripts, also regulates utrophin A post-transcriptionally by interacting with AREs located within the 3′-UTR. Given these two pathways, we sought to determine in the present work, the contribution of these two pathways to the overall regulation of utrophin A in muscle. Using a combination of experimental approaches, we discovered that miR-206 expression in cultured C2C12 cells and in dystrophic muscle fibers *in vivo* upregulates the endogenous utrophin A levels. This may appear surprising at first but we show, in additional experiments, that this upregulation of endogenous utrophin A is caused by the binding of miR-206 to conserved sites located in the 3′-UTR of KSRP, thus causing the subsequent inhibition of KSRP expression. This miR-206-mediated decrease in KSRP levels leads, in turn, to an increase in the expression of utrophin A transcript and protein due to a reduction in the activity of this destabilizing RNA-binding protein. Therefore, our study reveals that two apparent parallel and negative post-transcriptional pathways can have distinct impact, with miR-206 acting in these events as a molecular switch causing repression or activation of utrophin A expression by direct binding on the utrophin A 3′-UTR or via control of KSRP levels, respectively.

MicroRNA-206 is unique among the myomiR family because it is specifically found in skeletal muscle (MyomiR-1 and myomiR-133 are highly expressed both in skeletal and cardiac muscles) being either completely absent or expressed at relatively low levels in other tissues (11,12). Most of the research on miR-206 has so far focused on its function in skeletal muscle during differentiation using *in vitro* model systems (5,46,47). Importantly, a series of recent papers has shown that expression of miR-206 is altered in several neuromuscular disorders, indicating that miR-206 may also play a pivotal role *in vivo* in disease settings (18,26,48). Based on the available literature, it is clear that miR-206 is an important transacting factor capable of strongly repressing muscle gene and protein expression in skeletal muscle.

Similarly, cumulative evidence indicates that KSRP is an important post-transcriptional regulator of myogenic differentiation. In fact, several target mRNAs that are controlled by KSRP display rapid turnover rates in myoblasts and prolonged half-lives during differentiation (41,49). For example, Briata *et al.* (49) demonstrated that myogenin and p21 mRNAs are unstable in myoblasts and that KSRP is required for their decay. On differentiation of myoblasts into myotubes via serum withdrawal, phosphorylation of KSRP occurs and impairs its ability to interact with these ARE-containing transcripts, thereby leading to an increase in their stability (49,50).

In our work, we show that these two important transacting factors regulating muscle differentiation do in fact also interact in a post-transcriptional pathway that promotes repression of KSRP expression. We show that the 3′-UTR of KSRP contains functional miR-206 binding sites. Additionally, we demonstrate that increasing or decreasing miR-206 levels causes a reciprocal reduction or augmentation, respectively, in the abundance of KSRP in cultured myogenic and neuronal cells as well as in muscle fibers *in vivo*. Finally, we present data showing that expression of miR-206 is inversely correlated to that of KSRP during myogenic differentiation and muscle regeneration *in vivo*. Collectively, our findings thus show that KSRP is a direct target of miR-206 and, accordingly, provide key new insights into the mechanisms controlling skeletal muscle development. More specifically, the documented increase in miR-206 levels during differentiation and regeneration (18,20) may therefore be a prerequisite to cause repression of KSRP expression and subsequent enhanced stabilization of key myogenic transcripts. To our knowledge, our work represents the first demonstration that KSRP levels are reduced during myogenic differentiation.

The 3′-UTR of both mouse and human KSRP mRNA contains several additional conserved seed sequences for distinct miRNAs including Let-7, miR-27, miR-149, miR-181, miR-186, miR-485, miR-539, miR-543 and miR-882. In this context, a recent report has shown in host epithelial cells that miR-27b targets the 3′-UTR of KSRP to cause its translational repression, thereby modulating indirectly iNOS mRNA via ARE (51). Given these and our current findings, it would therefore be of interest to determine whether other miRNAs also modulate expression of KSRP not only in skeletal muscle cells but, additionally, in other cell types in which KSRP is known to have a prominent role such as in chondrocytes (52), P19 embryonal carcinoma cells (53), human embryonic kidney cells (54), differentiating N2a neuroblastomas (55) and inflammatory cells (56). This appears particularly warranted, as KSRP has been shown via a comprehensive microarray analysis performed with Hela cells, to target a set of 100 transcripts, thereby highlighting its importance as a master regulator of a plethora of mRNAs (57). Here, we establish a novel link between miR-206 and KSRP in these cell types. Our results complement several studies, which have previously demonstrated that KSRP is implicated in the machinery regulating the maturation of a cohort of miRNAs including miR-206, known to contribute to the modulation of different biological programs (58,59). Our data provide new insight on how KSRP acts as retro-control for miR-206, thereby playing an important role in the homeostasis of the skeletal muscle placed under different physiological situations such as regeneration and disease.

The 3′-UTR of mouse and human utrophin A mRNA contains a miR-206 binding site (29,30) and several AREs (39,60,61). Previous studies have shown that overexpression of miR-206 can repress expression of reporter constructs containing the 3′-UTR of utrophin A (29–31). In our recent work, we also identified that KSRP interacts with specific and conserved AREs located within the 3′-UTR of utrophin A mRNAs as to promote their decay (39). Given the existence of these two pathways, one could have predicted that both could act in parallel, perhaps even synergistically, to inhibit utrophin A expression. However, the miR-206 control of KSRP expression that we document in the current work promotes an increase in the endogenous expression of utrophin A. This is important, as utrophin A upregulation is currently envisaged as a potential therapy for DMD because it can functionally compensate for the absence of dystrophin along the sarcolemma of dystrophic muscle fibers. In support of our findings, miR-206 was shown to be highly expressed in mdx mouse muscle (18,20,27). Moreover, in agreement with our work, McCarthy *et al.* (27) have reported that expression of utrophin A is increased in dystrophic muscle in a manner that is not consistent with a direct regulation by miR-206, as proposed in existing literature.

The apparent discrepancy between previous work focusing on the miR-206-mediated downregulation of utrophin A with reporter constructs and the current findings can be reconciled if one considers the experimental systems used to obtain the divergent findings. It would appear that on overexpression of reporter constructs containing the utrophin A 3′-UTR, miR-206 is capable of targeting these reporter mRNAs to cause their repression. In more physiologically relevant conditions where one expects to see lower levels of expression of endogenous utrophin A mRNAs compared with overexpressed reporter constructs, the impact of miR-206 is greater on endogenous KSRP causing a decrease in its expression with a subsequent increase in utrophin A expression due to enhanced stabilization of its transcript. This regulatory mechanism highlights that the impact of important post-transcriptional regulators on expression of key mRNAs and proteins in skeletal muscle cells may vary significantly according to the cellular environment and the implicit fine balance between the relative abundance of these transacting factors and target mRNAs. In such a scenario, miR-206 can thus oscillate between direct repression via the 3′-UTR of utrophin A versus activation of its expression through an indirect pathway involving KSRP and AREs located within the 3′-UTR of utrophin A.

It seems important to note that this overlapping regulatory model implicating distinct post-transcriptional pathways is supported by findings of Vasudevan *et al.* (62) who reported that miR-369-3 regulation of target mRNAs can alternate between repressive and activation modes. In fact, another study has also shown that miR-466l upregulates IL-10 expression in TLR-triggered macrophages by competitively binding to the AREs of the IL-10 3′-UTR and thus, antagonizing the RNA-binding protein tristetraprolin-mediated IL-10 mRNA degradation (63). Collectively, these studies illustrate that miRNAs can indirectly affect expression of target transcripts by regulating the availability of other proteins also involve in regulating the same transcripts. Such regulatory loops indicate that the impact of miRNAs on mRNA and protein expression need not to be studied only in the context of miRNA–mRNA interactions but also, in the broader perspective of additional interactions of miRNAs with mRNA targets whose translation products, such as RNA-binding proteins, can also markedly influence the expression of the same target mRNA.

Over the last several years, converging lines of evidence indicate that miR-206 can play a pivotal role in promoting muscle regeneration, thereby impacting on the progression of neuromuscular disorders (19,20,48). Moreover, loss of miR-206 delays muscle regeneration in wild-type mice and exacerbates the dystrophic phenotype in mdx animals (18). In amyotrophic lateral sclerosis, a neurodegenerative disease characterized by loss of motor neurons and degeneration of target muscle fibers, miR-206 can delay progression of the phenotype by stimulating compensatory regeneration of neuromuscular synapses through targeting of histone deacetylases (HDAC4) and fibroblast growth factors (48). The positive impact of miR-26 on muscle regeneration and in these neuromuscular conditions likely occurs through the control of multiple target mRNAs (18–20,22–24,44,48,64). Taken together with the results of the present study showing that miR-206 controls the expression of utrophin A in skeletal muscle fibers, these findings suggest that this miRNA is clearly a therapeutic target of great potential for DMD.

## SUPPLEMENTARY DATA

Supplementary Data are available at NAR Online.

## FUNDING

Canadian Institutes of Health Research (CIHR) and the Association Française contre les Myopathies (AFM); Canada Research Chair in RNA Metabolism funded through the CIHR (to J.C.); Fellowship from the AFM during the course of this work (to A.A.). Funding for open access charge: CIHR.

*Conflict of interest statement*. None declared.

## Supplementary Material

Supplementary Data
