# Supplementary material for: Converging pathways involving microRNA-206 and the RNA-binding protein KSRP control post-transcriptionally utrophin A expression in skeletal muscle
Source: Nucleic Acids Res. 2013 Dec 26;42(6):3982–97. doi: 10.1093/nar/gkt1350 (PMC3973319; doi:10.1093/nar/gkt1350)
Supplement: Supplementary Data [file supp_42_6_3982__index.html]

Converging pathways involving microRNA-206 and the RNA-binding protein KSRP control post-transcriptionally utrophin A expression in skeletal muscle — Supplementary Data 

# Converging pathways involving microRNA-206 and the RNA-binding protein KSRP control post-transcriptionally utrophin A expression in skeletal muscle

## Supplementary Data

files

**Files in this Data Supplement:**

- Supplementary Data - docx file
- Supplementary Data - tif file
- Supplementary Data - tif file
