# Supplementary material for: Converging pathways involving microRNA-206 and the RNA-binding protein KSRP control post-transcriptionally utrophin A expression in skeletal muscle
Source: Nucleic Acids Res. 2013 Dec 26;42(6):3982–97. doi: 10.1093/nar/gkt1350 (PMC3973319; doi:10.1093/nar/gkt1350)
Supplement: Supplementary Data [file supp_gkt1350_nar-01959-a-2013-File010.docx]

**Figure S1. MicroRNA-1 targets the utrophin A 3’UTR.**

A) Shows luciferase activity observed with the different reporter constructs containing the 3’UTR of utrophin A upon overexpression of pre-miR-1. C2C12 myoblasts were co-transfected with reporter constructs containing either the wildtype utrophin A full-length 3’UTR or the mutated version, together with pre-miR-1 or pre-ve. We have also used the PHRG4-3’UTR construct (empty vector) as a negative control. B) Represents the activity of the luciferase reporter containing the mouse utrophin A 3’UTR in C2C12 cells transfected with miR-1 inhibitor (100 nM) or negative control. Values are means ± SE (n = 3 per group in triplicate). ***, *P* < 0.001 relative to corresponding control (Fig. S1A and B: unpaired t-test).

**Figure S2. Expression of utrophin A during myogenic differentiation.** A) Representative Western blots showing utrophin A levels in myoblasts (MB) and myotubes at day 1, 3 and 5 of differentiation. B) Utrophin A levels were quantified and are expressed as a percent of the levels seen in myoblasts. ß-actin served as loading control. Note that expression of utrophin A under these conditions is inversely correlated to the expression of KSRP (compare to Fig. 6B). Values are means ± SE (n = 4 per group). ***, *P* < 0.001; relative to corresponding control (Fig. S2A and B: paired one-way ANOVA and Bonferroni as Post-hoc test; Fig. S2D: Unpaired one-way ANOVA and Bonferroni as Post-hoc test).
