# Supplementary figures and images for: Converging pathways involving microRNA-206 and the RNA-binding protein KSRP control post-transcriptionally utrophin A expression in skeletal muscle
Source: Nucleic Acids Res. 2013 Dec 26;42(6):3982–97. doi: 10.1093/nar/gkt1350 (PMC3973319; doi:10.1093/nar/gkt1350)

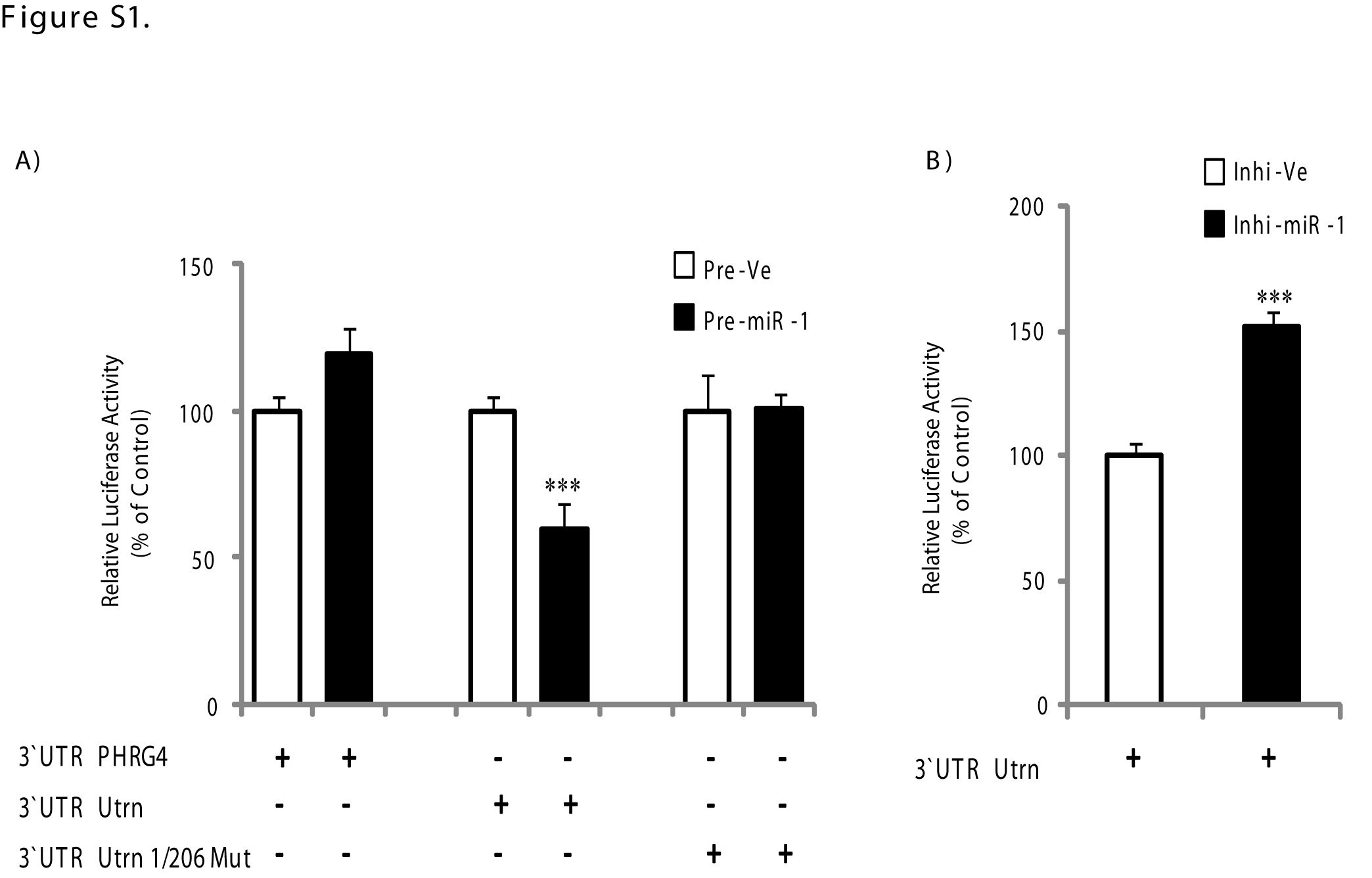

Supplement: Supplementary Data [file supp_gkt1350_nar-01959-a-2013-File011.tif]

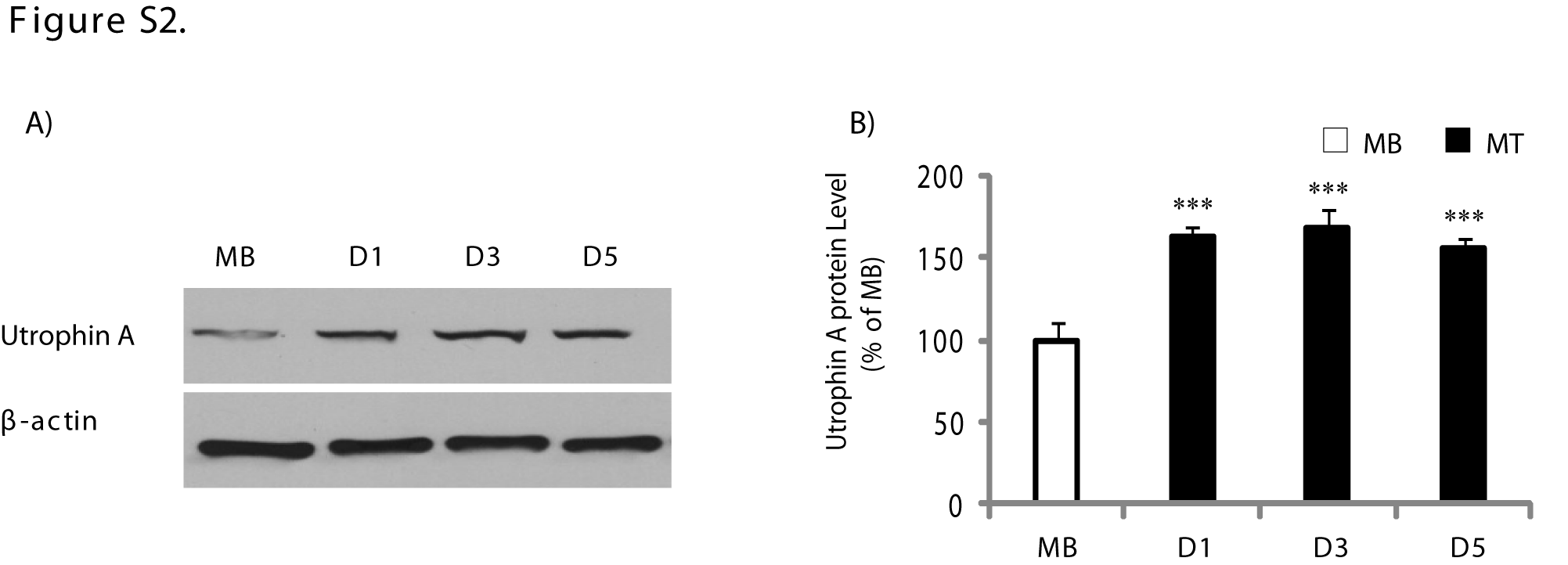

Supplement: Supplementary Data [file supp_gkt1350_nar-01959-a-2013-File012.tif]
